# Aging and cancer

**DOI:** 10.1186/s12943-024-02020-z

**Published:** 2024-05-18

**Authors:** Léa Montégut, Carlos López-Otín, Guido Kroemer

**Affiliations:** 1 Centre de Recherche des Cordeliers, Equipe labellisée par la Ligue contre le cancer, Inserm U1138, Université Paris Cité, Sorbonne Université, Paris, France; 2 Metabolomics and Cell Biology Platforms, Gustave Roussy Institut, Villejuif, France; 3 Facultad de Ciencias de la Vida y la Naturaleza, Universidad Nebrija, Madrid, Spain; 4 Institut du Cancer Paris CARPEM, Department of Biology, Hôpital Européen Georges Pompidou, AP-HP, Paris, France

**Keywords:** Age, Chemotherapy, Immunotherapy, Lifestyle, Modifiable risk factors

## Abstract

Aging and cancer exhibit apparent links that we will examine in this review. The null hypothesis that aging and cancer coincide because both are driven by time, irrespective of the precise causes, can be confronted with the idea that aging and cancer share common mechanistic grounds that are referred to as ‘hallmarks’. Indeed, several hallmarks of aging also contribute to carcinogenesis and tumor progression, but some of the molecular and cellular characteristics of aging may also reduce the probability of developing lethal cancer, perhaps explaining why very old age (> 90 years) is accompanied by a reduced incidence of neoplastic diseases. We will also discuss the possibility that the aging process itself causes cancer, meaning that the time-dependent degradation of cellular and supracellular functions that accompanies aging produces cancer as a byproduct or ‘age-associated disease’. Conversely, cancer and its treatment may erode health and drive the aging process, as this has dramatically been documented for cancer survivors diagnosed during childhood, adolescence, and young adulthood. We conclude that aging and cancer are connected by common superior causes including endogenous and lifestyle factors, as well as by a bidirectional crosstalk, that together render old age not only a risk factor of cancer but also an important parameter that must be considered for therapeutic decisions.

## Introduction

Aging is the most important risk factor of malignant disease, the prevalence of which dramatically increases as adults age, reaching a peak around 85 or 90 years, when the incidence of new cancer diagnoses starts to decline and that of cardiovascular and other diseases ramps up [1, 2]. Aging is, to some degree, modulable, meaning that chronological age (measured in years) and biological age (measured by biological tests and clinical status) can be uncoupled from each other [3, 4]. A young biological age is linked to a reduced risk of malignant disease [5, 6]. For this reason, it may even be argued - in a polemic fashion - that aging is a *modifiable* risk factor of cancer. This speculation is apparently supported by epidemiological data indicating that lifestyle factors that slow the aging process - such as leanness, an equilibrated mostly plant-based diet, voluntary physical activity and the avoidance of environmental mutagens - also reduce the probability to develop malignant disease [7, 8]. This observation suggests - but does not prove - that aging and cancer share common causes that are influenced by lifestyle or, in a slightly different vision, that manifest aging precipitates the development of clinically detectable tumors that then develop as ‘age-related diseases’.

**Fig. 1:**
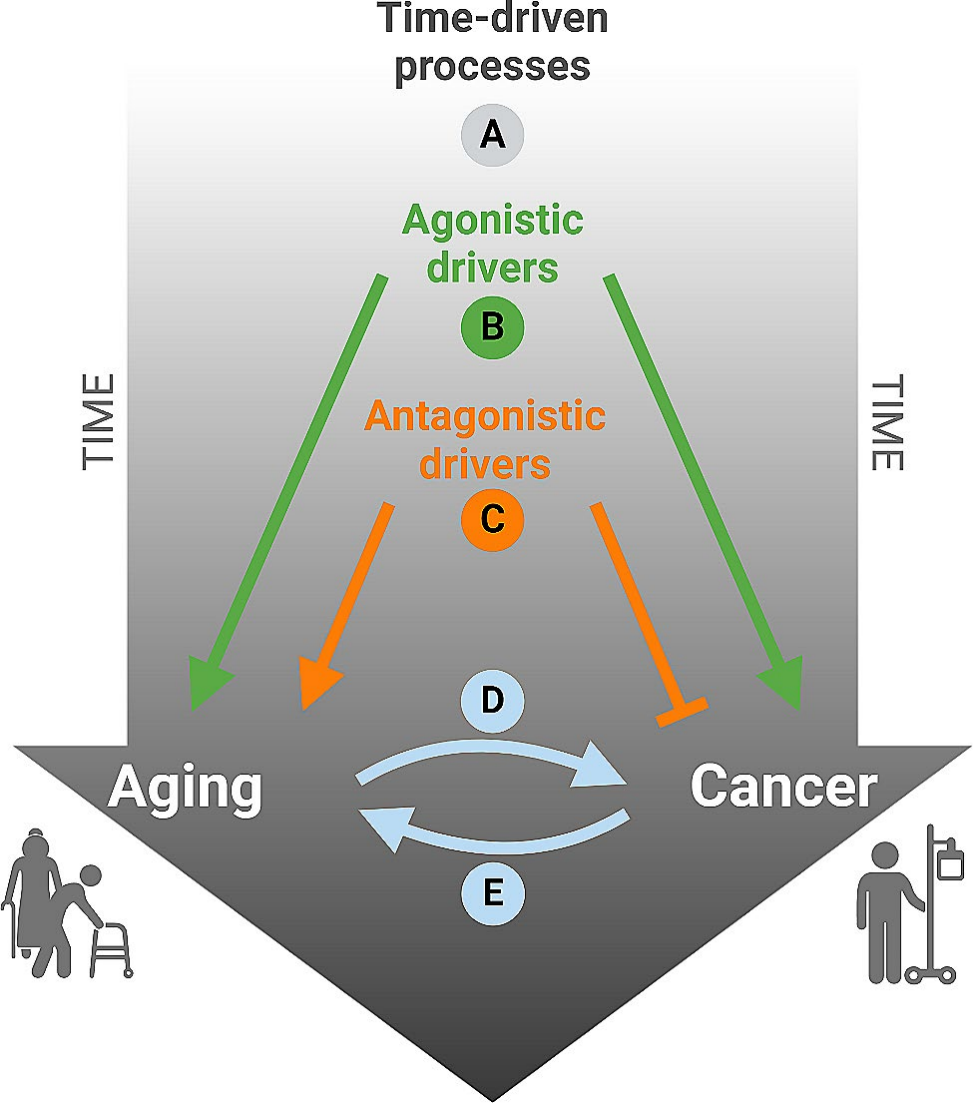
Potential relationship between aging and cancer. (**A**) Aging and cancer may lack a direct relationship and may rather be driven each independently by time (null hypothesis). (**B**) Agonistic drivers may cause aging and cancer in a time-dependent fashion. (**C**) Antagonistic drivers may favor aging while reducing the probability of carcinogenesis and tumor progression. (**D**) Aging tissues and organisms may be more prone for the development of cancers. (**E**) Cancer and its treatment may precipitate the deterioration of health and the aging process

In this review, we will examine the mechanistic connections between aging and malignant disease (Fig. 1). We will first discuss arguments in favor of the null hypothesis (Fig. 1A), namely, that aging and cancer just coincide as we become older because both are time-dependent processes but do not necessarily share a common biological basis. This null hypothesis would be in line with the existence of childhood cancers and progeroid (i.e., aging-accelerating) syndromes that do not increase the likelihood to develop cancer. We will then examine the likely more broadly applicable hypothesis that aging and cancer have common mechanistic grounds, as supported by the idea that both these processes share molecular and cellular characteristics that have been referred to as ‘meta-hallmarks’ or ‘agonistic hallmarks’ (Fig. 1B). However, this hypothesis does not explain why very old age (> 90 years) is accompanied by a reduction of the incidence of cancers, perhaps because certain ‘antagonistic hallmarks’ of aging counteract carcinogenesis (Fig. 1C). There is also the possibility that aged tissues are more susceptible to the development and clinical manifestation of cancers that then develop as a consequence of biological aging (Fig. 1D). Conversely, cancer and its treatment with chemotherapy and radiotherapy can precipitate aging, reducing healthspan and lifespan, as this is well documented for childhood cancer survivors (CCSs) as well as for survivors of cancers treated during adolescence and young adulthood (Fig. 1E). Finally, we will discuss the importance to weigh therapeutic decisions as a function of the oncological patient’s biological age.

## The null hypothesis: no causal links between aging and cancer

Although most malignancies manifest in older adults (> 65 years) [1, 2], there are specific cancers that are diagnosed during childhood or adolescence without any accompanying signs of accelerated aging or the simultaneous development of other age-associated disorders such as cardiovascular and neurodegenerative diseases. Such early cancers are comparatively rare (~ 1 in 5000 of the under 20-year-old, accounting for just 1% of all cancer diagnoses) and mostly manifest as non-epithelial malignancies (e.g., leukemias, central nervous systems cancers and lymphomas), contrasting with older adults that preponderantly develop carcinomas, and appear uncoupled from the aging process [9, 10]. Conversely, it can be argued that such early-life cancers (as exemplified by germ cell tumors, hepatoblastomas, medulloblastomas, neuroblastomas, osteosarcomas, retinoblastomas, rhabdomyosarcomas, and Wilms tumors) have a peculiar molecular etiology, distinguishing them from the tumors developing in older adults. In addition, each of these malignancies peaks at a different age (1–2 years for neuroblastoma, 3–4 years for Wilms tumor, 5 years for rhabdomyosarcoma…) suggesting an association with specific developmental stages rather than cumulative alterations that occur during classical (i.e., age-associated) oncogenesis. In any case, it appears that a specific subgroup of cancers is uncoupled from aging.

Dissociation of aging and cancer is also observed for specific progeroid syndromes, i.e., genetically disorders resulting in premature and accelerated aging [11]. In sharp contrast with several progeroid syndromes caused by defects in DNA repair (e.g. Bloom syndrome, Werner syndrome and Xeroderma pigmentosa, XP), which are linked to the early manifestation of cancers that often occur in an organ-specific fashion (e.g. leukemia and lymphoma in Bloom syndrome; thyroid cancer, skin cancer, and sarcoma in Werner syndrome; ultraviolet light-induced skin cancer in XP) [12–14], other progeroid syndromes are not associated with any type of early carcinogenesis. Thus, trichothiodystrophy, which is caused by mutations in genes that are also mutated in XP (*ERCC2*, *ERCC3*) and do not only compromise DNA repair (as this occurs in XP) but also impair transcription (as this does not occur in XP), is not associated with malignant disease [15]. Similarly, Cockayne syndrome, which is caused by mutations affecting the transcription-coupled repair branch of the nucleotide excision repair pathway (*ERCC6*, *ERCC8*), photosensitizes the skin (as this applies to XP as well) but does not cause causer, likely because mutated cells are eliminated before they can transform to a malignant state [16].

The dissociation of accelerated aging phenotypes and cancer also applies to defects in lamin A/C, e.g. Hutchinson-Gilford progeria syndrome (HGPS) due to mutations in lamin A encoded by *LMNA* or its protease STE24 encoded by *ZMPSTE24* [17]. As any other progeroid syndrome, HGPS causes segmental aging, i.e., an incomplete acquisition of aging phenotypes in only a few organ systems. Thus, HGPS patients develop some signs of aging (such as alopecia, wrinkled skin, osteoporosis, kidney failure, impaired vision and cardiovascular disease including atherosclerosis) but not others (such as cancer and neurodegeneration) during their infancy [18]. Similarly, Néstor–Guillermo progeria syndrome caused by *BANF1* mutations is associated with an aged appearance and skeletal abnormalities but not others (such as cancer, diabetes, cardiovascular and neurodegenerative diseases) [19]. Thus, several progeroid syndromes do not lead to an increase in the incidence of cancers. However, given the extreme rarity of these syndromes (e.g., 1 in 10 to 20 million children for HGPS), it may be argued that they constitute again ‘exceptions that confirm the rule’. Moreover, the premature death caused by progeria (i.e., usually before 20 years in HGPS due to cardiovascular disease), might ‘hide’ their pro-oncogenic potential.

In conclusion, there is evidence that, in rare instances, aging phenotypes and cancer development can be uncoupled from each other. This applies to specific progeroid syndromes that, however, cause incomplete (segmental) aging, as well as to a specific array of cancers developing in children and adolescents that are molecularly different from tumors developing in older adults.

## Common superior causes of aging and cancer

In contrast to the aforementioned exceptions, carcinomas, which constitute the most frequent category of cancers, as well as most glioblastomas, leukemias, lymphomas, melanomas and sarcomas, usually manifest at the age > 50 (in > 90% of all cases) and demonstrate a steady increase of incidence until the age of 85 years [1, 2]. Correlative evidence indicates that lifestyle factors that reduce biological aging also postpone or avoid the manifestation of cancer [20]. This applies to healthy lifestyles that increase organismal fitness including (i) a diverse, mostly plant-based diet based on natural ingredients (rather than highly processed foods, which are intrinsically toxic), avoiding overweight, obesity, hypovitaminoses, a deficit or surplus in oligoelements, as well as intestinal dysbiosis [21–23]; (ii) moderate or intense voluntary physical activity eluding excessive sedentarism, sarcopenia as well as osteoarthritis [24, 25]; (iii) avoidance of mutagenic toxins including excessive sun exposure, radiation, environmental poisons, air pollutants, tobacco and alcohol consumption [26, 27]; and (iv) psychosocial integration, which is often overlooked, yet essential for somatic health, in line with the fact that mental wellbeing and socioeconomic status are major determinants of healthspan, lifespan and the odds of cancer morbidity and mortality [28]. In accord with these observations, large epidemiological studies reveal that clinical factors for the most important age-associated ailments, i.e., cancer and cardiovascular disease, largely overlap [29, 30]. Of note, polygenic risk scores can predict the onset of both common cancers (such as mammary and prostate carcinoma) and cardiometabolic diseases [31].

**Fig. 2:**
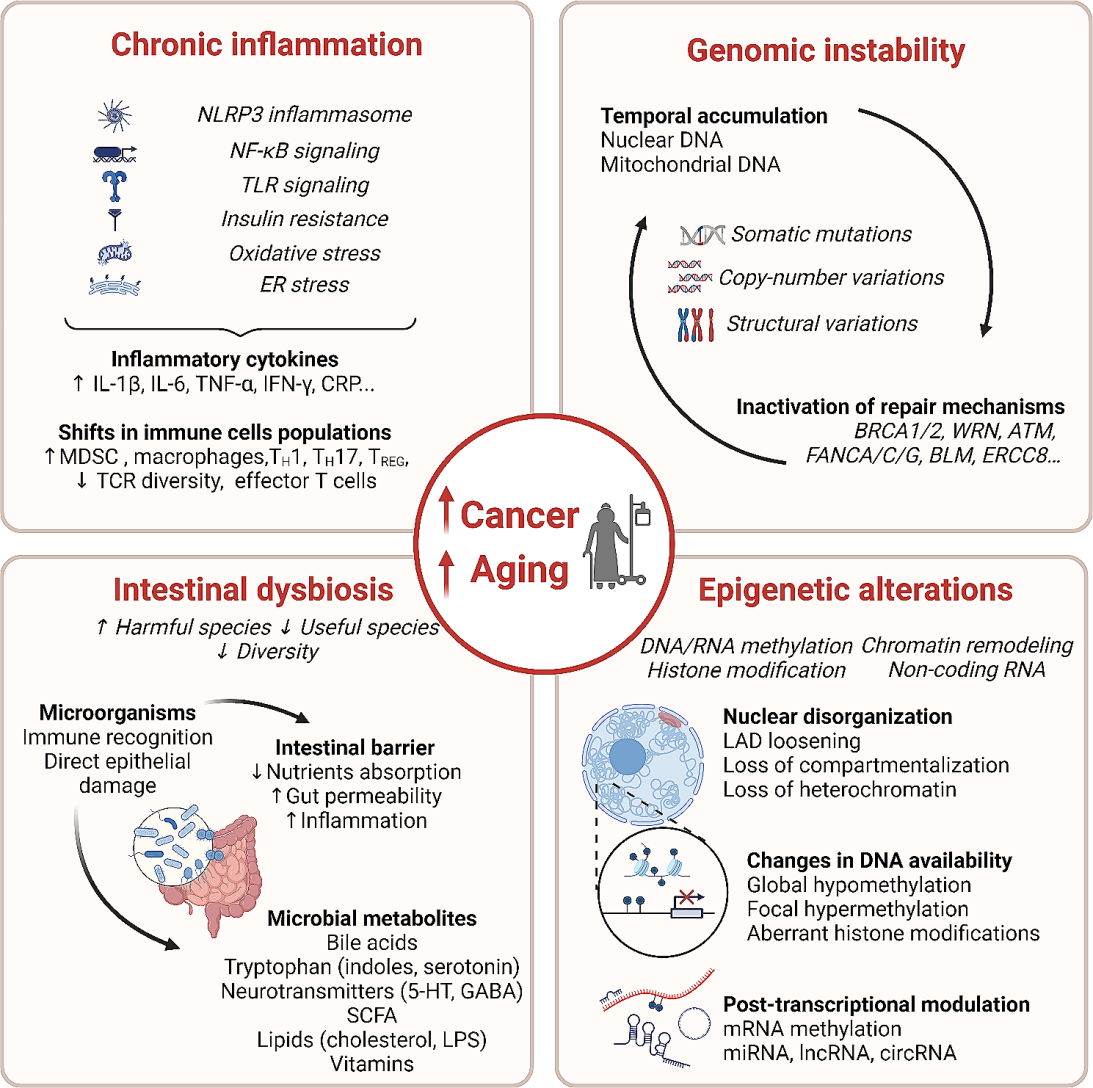
Common mechanisms driving cancer and aging. Cancer and aging are characterized by common hallmarks: the chronic installation of inflammation, genomic instability, intestinal dysbiosis and alterations of the epigenome. ATM: ATM serine/threonine kinase; BLM: BLM RecQ like helicase; BRCA1/2: Breast cancer type 1/2 susceptibility protein; CRP: circRNA: circular RNA; C-reactive protein; ER: endoplasmic reticulum; ERCC8: Excision Repair Cross-Complementing group 8; FANCA/C/G: FA complementation group A/C/G; GABA: Gamma-aminobutyric acid; IFN-γ: Interferon-gamma; IL: interleukin; LAD: lamina-associated domains; lncRNA: long non-coding RNA; LPS: lipopolysaccharide; MDSC: myeloid-derived suppressor cells; miRNA: micro RNA; mRNA: messenger RNA; NF-κB: Nuclear factor kappa-light-chain-enhancer of activated B cells; NLRP3: NLR family pyrin domain containing 3; SCFA: short-chain fatty acids; TCR: T cell receptor; T_H_: helper T cell; TLR: Toll-like receptor; TNF-α: Tumor necrosis factor; T_REG_: regulatory T cell; WRN: WRN RecQ like helicase; 5-HT: 5-hydroxytryptamine (Serotonin)

The aforementioned associations between aging, cancer and cardiovascular disease suggest - but do not prove - that these conditions are dictated by common superior causes. What are then the hypothetical pathways that link such overarching mechanisms of aging and cancer? Such pathways can be tentatively identified among the ‘hallmarks’ of aging [3] and cancer [32], which do not only accompany the relevant processes but also accelerate them if they are experimentally or accidentally induced and, on the contrary, decelerate, halt or reverse aging as they simultaneously prevent carcinogenesis if they are attenuated by genetic or pharmacological manipulations [3, 32]. Several hallmarks of aging (i.e., genomic instability, epigenetic alterations, chronic inflammation and dysbiosis) are also described as hallmarks of cancer and hence constitute common ‘meta-hallmarks’ or ‘agonistic hallmarks’ [33] (Fig. 2).

### Genomic instability

Mutations affecting chromosomal DNA occur spontaneously as well as in response to exogenous mutagens, resulting in a progressive, age-dependent accumulation of genomic alterations [34]. Next-generation sequencing of DNA extracted from circulating myeloid cells allows for the detection of clonal hematopoiesis of indetermined potential (CHIP). This alteration manifests with aging and constitutes a risk factor of blood cancers, including acute myeloid leukemia [35], as well as other seemingly unrelated diseases, such as atherosclerosis [36], liver fibrosis [37] and non-small cell lung cancer [38], likely due to pro-inflammatory effects. In recent years, it has been discovered that genomic instability affects all major organs, causing the generation of mosaics of cells (i.e., the juxtaposition of genetically non-identically cells within the same tissue), some of which tend to clonally expand because they acquire a proliferative advantage over normal, unmuted cells, hence outcompeting them [39, 40]. The resulting genetic heterogeneity may contribute to the time-dependent functional decline of aging tissues (for instance due to a final loss of stem cell features, replicative senescence, the secretion of pro-inflammatory factors) as well as the generation of ever-more mutated, pre-malignant and hence potentially oncogenic cells.

### Epigenetic alterations

The structure of chromatin and patterns of gene expression are transmitted through epigenetic changes which result from a myriad of posttranslational modifications (most prominently methylation and acetylation) affecting DNA and histones (along with other mechanisms involving non-coding RNAs), as well as chromatin structure, that can be transmitted from mother cells to their daughter cells, hence contributing to the “identity” of differentiated cell types [41]. Throughout the aging process, such epigenetic changes are progressively lost, increasing the noise in the system, and contributing to a progressive loss of cellular identities that menaces the functional integrity of complex tissues and potentially enhances the risk of carcinogenesis coupled to an increase in tumor heterogeneity and phenotypic plasticity [42]. The most common (but still imperfect) technology to measure epigenetic shifts consists in bisulfite pyrosequencing to detect DNA methylation patterns that can be bioinformatically deconvoluted as “biological clocks” and be associated to the risks of developing specific diseases [43].

### Chronic inflammation

Aging is associated with a failure to control inflammation in space and time (“inflamm-aging”) [44], and inflammation is also one of the hallmarks of cancer, likely acting through a combination of cell-autonomous effects (e.g., increased proliferation of cells leading to genomic and epigenomic instability) and non-cell-autonomous consequences (e.g., fibrosis, rarefaction of ECM components and local immunosuppression by myeloid-derived suppressor cells) [3, 45]. For this reason, inflammation has a dual role in both aging and cancer, implying that suppression of inflammation may have a multipronged impact on the development of a large spectrum of age-associated disorders that includes both malignant and non-malignant diseases.

### Intestinal dysbiosis

The intestinal lumen is colonized by a diverse microecosystem composed by archaea, bacteria, fungi, parasites, phages and viruses that altogether influences gut health as well as bodywide homeostasis [46]. Contrasting with the healthy (eubiotic) state, gut dysbiosis is characterized by an increase in the abundance of harmful microbial species coupled to a relative decrease of useful microbes. Importantly, multiple non-malignant age-associated diseases are coupled with similar shifts in the gut microflora as are cancers located outside of the gastrointestinal tract [47]. Experiments showing that the microbiota from young mice, as well as specific health-associated bacterial strains (such as *Akkermansia muciniphila*), can enhance the lifespan of mice with progeria, suggest a causal implication of dysbiosis in aging [48]. Intriguingly, the transfer of such a health-associated microbiota or that of *A. muciniphila* also stimulate anticancer immunosurveillance [49] (and fecal microbial transfer from healthy patients to melanoma-bearing patients sensitizes to subsequent immunotherapy with antibodies targeting the PD-1/PD-L1 interaction) [50], suggesting communalities between the age-related loss of health and cancer. Thus, intestinal dysbiosis is considered another ‘meta-hallmark’ of aging and cancer.

In sum, it appears that some of the processes that cause aging also underly oncogenesis, as this is well documented for the accumulation of mutated cells in the aging organism, likely preparing the grounds for multi-step oncogenesis, as well as for the loss of epigenetically controlled cellular identities that may favor the acquisition of cancer stem cell characteristics. Chronic inflammation and dysbiosis also share similar etiologies and trajectories in the context of aging and cancer with the peculiarity that they can be targeted by specific treatments.

## Possible causes of reduced cancer incidence in very old people

**Fig. 3:**
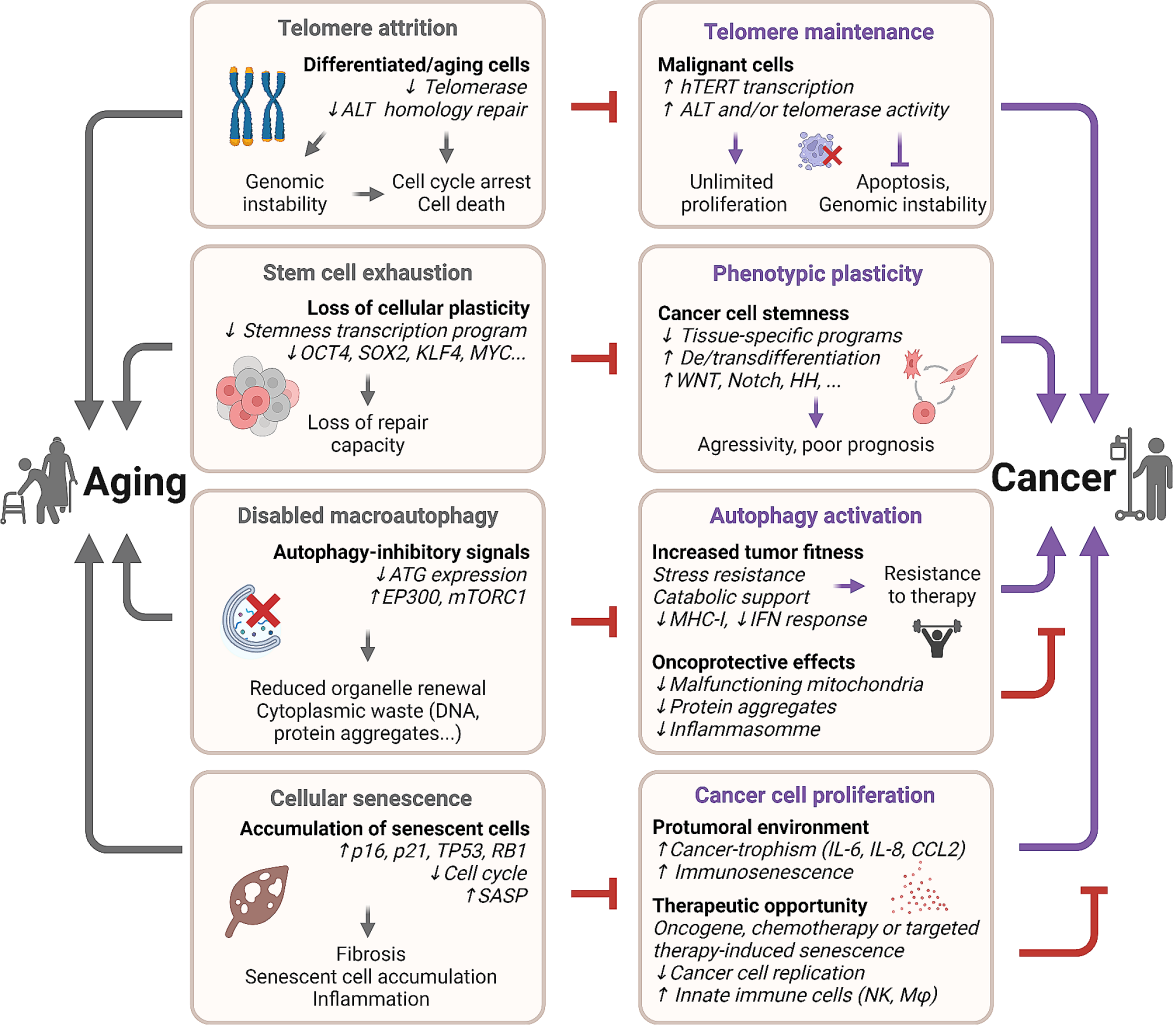
Mechanisms of aging that oppose cancer development. Part of the aging phenotype results in the blockade of mechanisms that typically sustain tumor development and growth. Telomere attrition, stem cell exhaustion, disabled macroautophagy and cellular senescence are increased in aging and have an antagonist role in cancer. ALT: Alternative lengthening of telomeres; ATG: autophagy-related genes; CCL2: chemokine C-C motif ligand 2; EP300: histone acetyltransferase p300; HH: hedgehog signaling pathway; hTERT: Telomerase reverse transcriptase; IFN: interferon; IL: interleukin; KLF4: Kruppel-like factor 4; MHC-I: major histocompatibility complex class I; mTORC1: mammalian target of rapamycin complex 1; Mφ: Macrophage; NK: natural killer cell; Notch: neurogenic locus notch homolog proteins signaling pathway; OCT4: octamer-binding transcription factor 4; p16: cyclin-dependent kinase inhibitor 2 A; p21: cyclin-dependent kinase inhibitor 1; RB1: Retinoblastoma protein; SASP: senescence-associated secretory phenotype; SOX2: sex determining region Y-box 2; TP53: tumor protein P53; WNT: Wnt signaling pathway

Nonagenarians (90–99 years), centenarians (100–109 years) and supercentenarians (> 110 years) progressively exhibit a relative decrease in the incidence of new cancer diagnoses as compared to the younger octogenarians (80–99 years) and septuagenarians (70–79 years) [1, 2], suggesting that some facets of the aging process may protect against the development and clinical manifestation of neoplasia. Indeed, the probability of a centenarian to die from cancer as opposed to other causes is only 4% [51]. Specific features of aging (i.e., telomere attrition and stem cell exhaustion) can suppress oncogenesis and hence act as ‘antagonistic’ hallmarks. Disabled macroautophagy and cellular senescence are two additional ‘ambivalent’ hallmarks of aging that mediate context-dependent oncosuppressive effects [33] (Fig. 3).

### Telomere attrition

Telomeres at the extreme ends of chromosomes contain repeated sequences that must be maintained by the telomerase complex to avoid their progressive shortening during mitoses. Since telomerase subunits are typically lost during adulthood and aging in somatic cells, this mechanism limits replicative lifespan and potentially contributes to the aging process as a countdown mechanism [52]. Telomere attrition theoretically avoids carcinogenesis in aged tissues due to the induction of replicative senescence, and tumors must indeed re-activate telomerase expression (e.g., due to mutations in the promoter encoding the protein subunit TERT) [53], overexpress additional factors (such as the shelterin compound TPP1) that cooperate with telomerase in telomere maintenance [54], or activate mechanisms for alternative lengthening of telomers to strive [55].

### Stem cell exhaustion

Stem cell exhaustion compromises tissue repair in aging [56, 57]. Although this has negative effects on the capacity of tissues to regenerate upon injury, stem cell exhaustion may also prevent oncogenesis by opposing phenotypic plasticity and hence reduce the probability of malignant transformation in aged tissues [33]. In other words, stem cell exhaustion can abort the first steps of oncogenesis, which relies on the formation cancer stem cells. Indeed, malignant transformation implies a failure of normal terminal differentiation by cells that rather undergo de-differentiation, manifest a differentiation block or exhibit transdifferentiation [32]. Some of these pathways related to phenotypic plasticity (such as signals transmitted via Wnt/β-catenin, NF-κB, Hedgehog) are explored in clinical trials [58] and Smoothened (Smo) antagonists can be targeted for the treatment of locally advanced and metastatic basal cell carcinoma [59], underscoring the practical relevance of these findings.

### Disabled macroautophagy

Aging is associated to a progressive inhibition of macroautophagy (and other types of autophagy, including chaperone-mediated autophagy and mitophagy), progressively compromising cellular fitness due to the accumulation of waste material including dysfunctional organelles and micronuclei [60, 61]. Disabled macroautophagy may also compromise the fitness of cancer cells, reducing their metabolic fitness, proliferative potential, resistance to therapeutic agents, as well as their capacity to subvert anticancer immune responses [33]. That, said, macroautophagy may also constitute a tumor-suppressive mechanism because it contributes to the maintenance of genomic stability, favors oncogene-induced senescence, mitigates procarcinogenic inflammation, contributes to ferroptotic cell death [62] and favors immunosurveillance [33, 63]. Hence, it appears that macroautophagy plays a context-dependent role, either favoring or inhibiting oncogenesis and tumor progression.

### Cellular senescence

Senescent cells exhibiting accumulate in aging tissues, and it has been postulated that their close-to-irreversible cell cycle arrest would constitute a barrier against malignant transformation [33]. Accordingly, the induction of senescence in malignant cells may constitute a therapeutic goal, especially since senescent cancer cells appear to be particularly immunogenic, hence eliciting T cell responses via the upregulation of the antigen-presenting machinery in response to interferon-g [64, 65]. In addition, senescent tumor cells appear particularly susceptible to natural killer (NK) cell-mediated lysis [66, 67]. However, senescence may be reversible in specific cases, a phenomenon that might contribute to tumor cell dormancy [68, 69]. Moreover, senescence can result in local immunosuppression due to upregulation of the two PD-1 ligands PD-L1 and/or PD-L2 on malignant cells [70, 71], as well as in the secretion of pro-inflammatory and immunosuppressive factors exemplified by interleukins 6 and 8 [72]. This latter phenomenon, which is dubbed as senescence-associated secretory phenotype (SASP), explains the long-range effects of cellular senescence [73]. When senescence affects tumor-infiltrating leukocytes, it may subvert anticancer immune responses (see below), hence contributing to tumor progression. Furthermore, senescence affecting stromal cells (such as hepatic stellate cells in the context of hepatocellular carcinoma) may precipitate oncogenesis [74]. For this reason, senescence mediates context-dependent anti- and pro-carcinogenic effects.

In sum, several among the hallmarks of aging may reduce the generation or fitness of (pre-)malignant cells, likely explaining why the oldest elderly exhibit a reduced cancer-specific mortality. That said, although such aging-associated tumor suppressive effects may have a significant impact on cancer development in very old persons, they fall short from reducing cancer incidence to the levels found before 20 years of age [1, 2].

## Cancer as a complication of aging

**Fig. 4:**
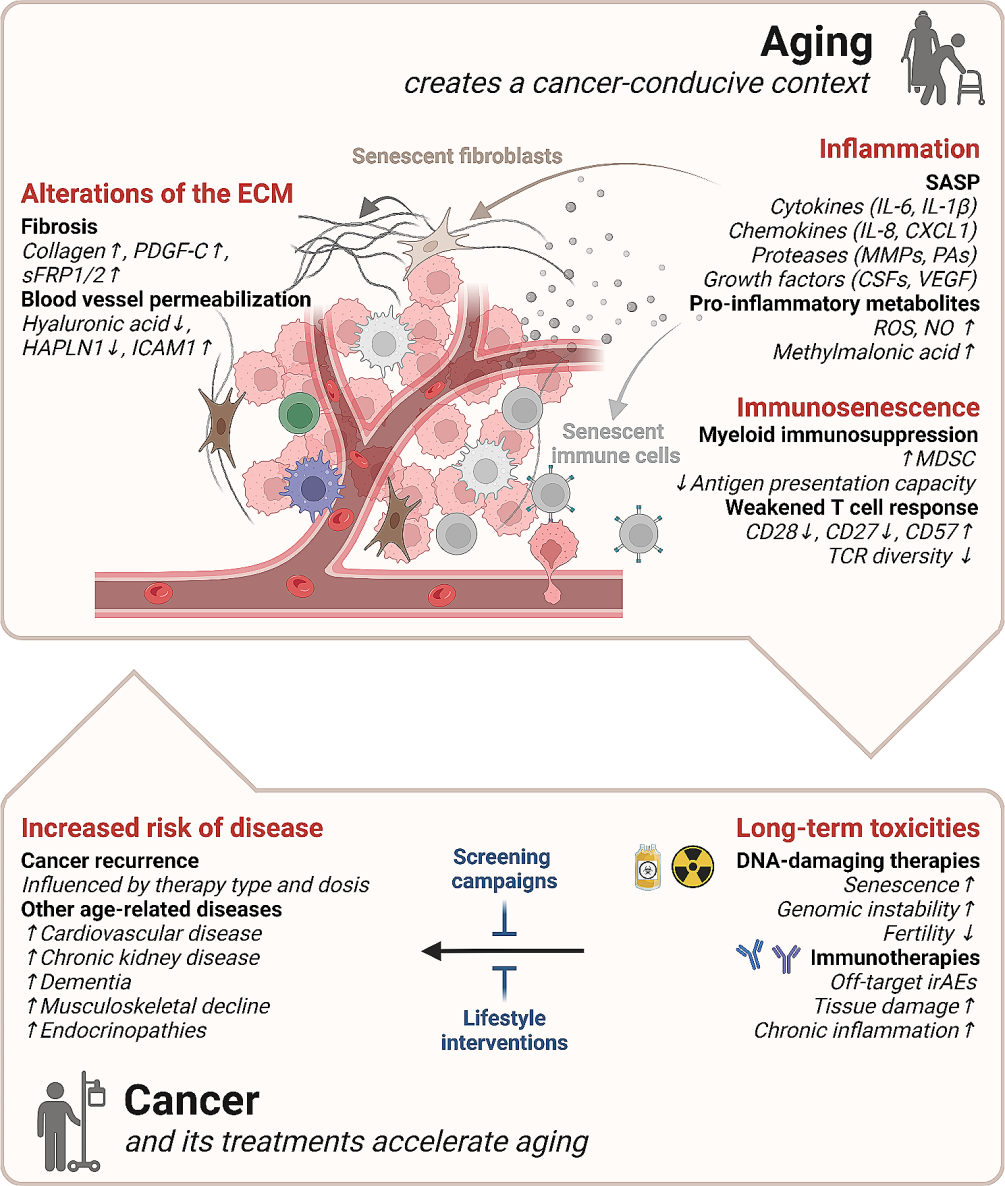
Reciprocal induction of aging and cancer The aged organism is particularly propitious for the development of malignancies due to alterations in the extracellular matrix (ECM) and the installation of a favorable immune context (inflammation and immunosenescence). Conversely, after their curative treatment cancer survivors face long-term toxicities including accelerated aging. Indeed, in the long run, they have higher probabilities of cancer relapse as well as increased risk of developing a plethora of age-related pathologies. CD: Cluster of differentiation; CSF: colony-stimulating factors; CXCL1: chemokine (C-X-C motif) ligand 1; FRP1/2: secreted frizzled-related proteins 1/2; HAPLN1: hyaluronan and proteoglycan link protein 1; ICAM1: intercellular adhesion molecule 1; IL: interleukin; irAEs: immune-related adverse events; MDSC: myeloid-derived suppressor cells; MMPs: matrix metalloproteinases; NO: nitric oxide; PA: protease associated domain proteins; PDGF-C: platelet-derived growth factor C; ROS: reactive oxygen species; TCR: T cell receptor; VEGF: vascular endothelial growth factor

The aforementioned considerations suggest that cancers steadily increase their frequency in the aged organism until the plateau reached at 85–90 years is attained, because aging and oncogenesis are caused by shared mechanisms (and simultaneously other aging-driving processes fail to avoid carcinogenesis). However, it can also be speculated that age-associated changes in tissue quality with fibrosis and alterations of the extracellular matrix (ECM), systemic and local inflammation, as well as failure of immunosurveillance favor carcinogenesis and tumor progression [75, 76]. Hence aging itself (rather than its underlying causes) would support the clinical manifestation and progression of cancers as a secondary complication of aging (Fig. 4).

### Alterations of the extracellular matrix

Aging is associated to the development of fibrosis due to the excessive deposition of ECM components such as collagen in the ECM in several internal organs. This property may explain why aging is coupled to an increased propensity of breast cancers and melanomas to generate metastases in the lung. Indeed, in preclinical experiments, fibrosis of the lung causes the reversal of dormancy of cancer cells via the fibroblast-mediated secretion of platelet-derived growth factor (PDGF)-C (in the case of estrogen receptor-positive breast cancer) or that of WNT antagonist, sFRP1 (in the case of melanoma) [77, 78]. Reportedly, aged dermal fibroblasts also secrete high levels of another WNT antagonist, sFRP2, which can drive angiogenesis in melanomas, their metastasis, as well as their resistance to targeted therapy with the BRAF inhibitor vemurafenib [79]. In addition, age-associated disruption of the collagen I network in the ECM of the dermis may reduce mechanical constraints that prevent the development of basal cell carcinoma [80].

An age-related decrease in the secreted ECM polysaccharide hyaluronic acid, especially in its high-molecular mass variant, may causally contribute to aging and oncogenesis, as demonstrated by the fact that transgenic mice overexpressing naked mole-rat hyaluronic acid synthase 2 gene exhibit an increase in cancer-free healthspan and longevity [81]. This age-associated decrease in hyaluronic acid, as well as that of the proteoglycan link protein hyaluronan and proteoglycan link protein 1 (HAPLN1), may induce an aging-associated increase in ICAM1 in endothelial cells [82]. ICAM1 overexpression causes phosphorylation and internalization of VE-cadherin, resulting in blood vessel permeabilization, potentially explaining why old age is associated with poor melanoma outcome. Indeed, blocking ICAM1 with suitable antibodies reduces tumor size and distant metastasis in older mice with melanoma [82].

### Inflammation

Inflammaging [44] can drive the senescence of cancer-associated fibroblasts that secrete factors enhancing peritoneal dissemination of gastric cancer [83]. Gliosis, a state of central nervous system inflammation coupled to the expansion of glial cells (such as microglia and astrocytes causing microgliosis and astrogliosis, respectively, during early and late responses to injury) promotes metastasis of lymphoma to the brain due to the upregulation of the chemokine CCL19, locally retaining tumor cells [84]. As compared to plasma from young controls, plasma from aged individuals contains higher levels of methylmalonic acid, a byproduct of propionate catabolism and a biomarker of vitamin B12 deficiency [85]. B12 deficiency may favor inflammation indirectly through a failure in tissue repair [86]. Of note, methylmalonic acid favors epithelial-mesenchymal transition of cancer cells through the upregulation of TGFB2 and consequent upregulation of the transcription factor SOX4 [85]. In addition, methylmalonic acid has pro-inflammatory and pro-aging properties [87]. These examples illustrate how age-associated inflammation favors tumor progression.

### Failing immunosurveillance

Aging of the immune system (immunosenescence) occurs in the elderly, thus compromising anticancer immune response that may avoid carcinogenesis, reduce tumor progression, and decisively contribute to the success of most if not all treatment modalities in the oncological armamentarium, including chemotherapy, radiotherapy, immunotherapy and targeted therapy [88, 89]. Immunosenescence may directly affect T cells, reducing their effector function by down-regulating the costimulatory markers CD28 and CD27 and upregulating the terminal differentiation marker CD57 [90, 91]. In addition, senescent macrophages accumulate in tissues such as the lung, facilitating KRAS-induced non-small cell lung cancers, likely due to direct trophic effects on malignant cells, as well as due to the suppression of T cell-mediated immunosurveillance. Accordingly, the elimination of senescent macrophages reduces tumor progression [92, 93]. Failing immunosurveillance may also contribute to aging due to the incapacity of the immune system to clear senescent cells that accumulate in various tissues. Logically, attempts are underway to stimulate immune responses against such senescent cells, for instance by engineering chimeric antigen receptor (CAR) T cells that recognize antigens associated with cellular senescence [94, 95].

In conclusion, the aging organism appears particularly susceptible to the development and progression of malignant tumors through a variety of mechanisms. Aging tissues may constitute a particularly appropriate ‘soil’ for tumors to seed and invade.

## Aging as a consequence of cancer and its treatment

Invasive cancers break tissue barriers, cause chronic inflammation, suppress immune responses, and mobilize ever more resources from the body, ultimately eroding bodywide health at multiple levels [96]. Moreover, even when successful, their treatment with DNA-damaging chemotherapeutics and radiotherapy has long-lasting effects on the organism that may manifest with a delay of several decades in cancer survivors cured during childhood, adolescence, or young adulthood. These long-term consequences give rise to a premature aging phenotype coupled to the early manifestation of a large panel of age-associated pathologies that include, but are not limited to, the manifestation of other (‘subsequent’ or ‘second’) cancers, small adult height, prediabetes, cardiovascular disease, chronic kidney disease, dementia, musculoskeletal decline with osteoporosis and sarcopenia, as well as tissue fibrosis. Ultimately, this results in frailty and early mortality (Fig. 4). These long-term complications of early-life cancer treatments have been described in some detail thanks to the constitution of specific registries such as the St. Jude Lifetime Cohort [97, 98], the US-centered Childhood Cancer Survivor study [98, 99], and the EUROCARE-6 study [100]. Although not as obvious as observed in childhood cancer, the additive burden of previous cancer in terms of chronic pathologies and premature mortality can be calculated in the adult population. Using data from the UK biobank, the health data from over 240,000 cancer survivors was compared to that of 500,000 adults with no history of cancer after matching by age, sex, and Index of Multiple Deprivation. Late morbidities attributable to cancer included hematological, pulmonary, Immune and renal dysfunctions, and depended on the type, doses and combination of used therapies [101]. Logically, attempts are underway to palliate these undesired side effects by more appropriate treatments reducing long-term toxicity, screening programs that identify patients at risk of developing specific diseases, as well as by post-therapeutic lifestyle interventions.

### Avoidance of long-term toxicities of anticancer treatments

There is clear evidence that the severity of the age- and disease-accelerating effects of early-life cancer therapies have diminished over time likely due to several factors including, but not limited to, the reduction of cumulative chemotherapy doses, the replacement of some DNA-damaging agents by other cytotoxicants, and the avoidance of certain interventions, such as cranial irradiation of children with leukemia or glioma; or mediastinal irradiation of patients with Hodgkin lymphoma [102, 103]. Retrospective analyses identifying risk-enhancing practices and biomarkers may help to reduce treatment-induced long-term toxicities in prospective studies. Thus, telomere length in circulating lymphocytes is reduced in CCSs, correlating with the manifestation of a variety of non-neoplastic chronic health conditions [104]. Similarly, the measurement of various signs of biological aging (two physiology-based algorithms; four distinct DNA methylation clocks, and a single-time-point DNA methylation blood test) revealed that CCSs from the St. Jude Liftetime cohort aged more quickly (by ~ 5% in average) than community controls, in particular when they received hematopoietic cell transplants and vinca alkaloid chemotherapy [105]. Although these quantitative tests are predictive of mortality [105], it remains to be determined whether such biomarkers may guide the development of less toxic cancer cures.

An additional strategy consists in the use of co-medications that can reduce anticancer drug toxicities. For example, co-treatment with the iron chelator dexrazoxane has been successfully used to mitigate the long-term side effects of anthracyclines at the level of serious cardiovascular outcomes (cardiomyopathy, ischemic heart disease, and stroke) in CCSs [94, 95]. Moreover, in a randomized Phase II trial, low-dose tamoxifen has been shown to reduce radiological and biological risk factors of breast cancer in patients having received chest radiation ≥ 12 Gy by the age of 40 [106]. Anthracycline-induced premature aging can be prevented in mice by a chemical-genetic system that allows for the elimination of senescent cells [107]. Hence, senolytics, which are drugs that kill senescent cells, can be used to combat the long-term cardiotoxicity of doxorubicin in a preclinical model [108]. Future will tell whether such an approach can also be used to mitigate therapy-induced senescence in cancer patients as well.

### Biomarker-guided screening programs

The risk of subsequent (secondary) cancers can be calculated based on polygenic risk scores derived from general population and genome-wide association studies [109]. Moreover, this risk is influenced by the type of treatment (radiotherapy and specific chemotherapeutic agents) and their cumulative doses [110]. More than cumulative interactions between genetic risk and radiotherapy have been described for specific cancers such as basal cell carcinoma and breast or thyroid cancers [109]. The use of doxorubicin beyond a threshold (≥ 200 mg.m^-2^) is linked to an enhanced risk of subsequent female breast cancer [110], exemplifying how distinct therapeutic interventions are linked to particular cancer risks that may instigate a reinforcement of early detection campaigns. Among CCSs, hearing loss is associated with the use of cisplatin, carboplatin and cranial or facial radiation > 32 Gy [111]. The risk of cardiac failure is determined by cumulative anthracycline doses and the location of radiotherapyn e.g., targeting the mediastinum causing irradiation of the heart [112, 113], while the risk of severe obesity in CCSs is influenced by genetic risk scores [114]. Thus, particular features of early-life cancer therapy may be combined with polygenic risk scores to guide specific screening programs for the detection and interception of specific manifestations of premature aging in CCSs.

### Lifestyle interventions

Retrospective studies indicate that premature aging of CCSs is reduced by enhanced uptake of dark green vegetables and nuts/seeds, but enhanced by that of refined grain [115]. In contrast it appears that physical activity has no significant impact on the probability of CCSs to develop subsequent cancers [116, 117]. However, physical activity in adult CCSs has been shown to correlate with reduced neurocognitive problems at the levels of emotion regulation, memory, organization and task efficiency [118], as well as with reduced mortality [116]. In addition, psychosocial stress, sleep perturbations, smoking, alcohol consumption and substance use may contribute to accelerated ageing in CCSs [119] in the same way as they deteriorate health in cancer-free individuals [28]. These findings suggest that lifestyle factors that favor health in the general population may also be useful for maintaining the fitness of CCSs.

In sum, survivors of early-life cancer exhibit accelerated aging with the precocious manifestation of age-associated diseases, as well as an elevated risk of frailty and premature death. Attempts are underway to reduce these risks. Thus, secondary prevention in childhood cancer survivors is constantly ameliorated following specific guidelines, such as the Children’s Oncology Group Long-Term Follow-Up Guidelines for Survivors of Childhood, Adolescent, and Young Adult Cancers in North America [120], the Pan-European Network for Care of Survivors after Childhood and Adolescent Cancer Guidelines Group [121], as well as the International Guideline Harmonization Group for Late Effects of Childhood Cancer [122]. Beyond these risk reduction programs, efforts are ongoing to implement lifestyle interventions that reduce accelerated aging in cancer survivors. It will be interesting to learn whether drugs that are currently evaluated for their potential antiaging effects in clinical trials [4] can be advantageously used in cancer survivors as well.

## Impact of aging on the therapeutic management of cancer

The classification of cancer is still mostly based on location (organs) rather than on molecular subtypes. When classified by location or histology, the prognosis of each cancer type changes with age [123]. For example, breast cancers tend to be particularly aggressive if they manifest before 40 years of age [124], while Hodgkin lymphoma diagnosed after 45 has a dismal prognosis compared to cases diagnosed in adolescence or early adulthood [125]. Similarly, the efficacy of treatments regimens changes with age. For instance, oxaliplatin fails to confer any benefit for the adjuvant treatment of poor-prognosis colorectal cancer after the age of 70 [125], but immunotherapy against melanoma or lung cancer is equally efficient at a young and an old age [126, 127]. Considering that adjuvant or neoadjuvant chemotherapy of breast cancer patients leads to a 10% reduction of exercise capacity, measured by oxygen uptake during peak exercise (VO_2peak_), and that normal aging is accompanied by a 10% reduction of VO_2peak_ per decade, the age acceleration induced by therapeutic interventions on adult patients is certainly problematic [128]. For this reason, generic recommendations such as the avoidance of chemotherapy and a preference for radiotherapy for the management of older cancer patients have been proposed [129]. However, this idea collides with the fact that the suppression of chemotherapy in older breast cancer patients is associated with an elevated risk of relapse [130].

The majority of cancers manifest in older adults (> 65 years), often in the context of advanced biological age (with respect to chronological age) and one or several comorbidities. This contrasts with the fact that most clinical trials are performed in younger, relatively fit individuals, because they usually exclude persons > 70 years with major comorbidities and reduced performance status [131, 132], meaning that FDA/EMA-approved treatments are often not adapted to the average ‘real world’ cancer patient. Indeed, older adults diagnosed with cancer may exhibit more side effects and reduced drug tolerability than patients enrolled in clinical trials. For this reason, it is necessary to carefully weight therapeutic decisions to avoid the over-treatment or under-treatment of older patients. Over-treatment consists in surgical procedures or the administration of excessive doses (of drugs or irradiation) or cycles of treatments, resulting in a reduction of the quality of life without therapeutic benefit, as this often occurs near the end of life in older patients [133]. Under-treatment consists in the exclusion of older patients from viable therapeutic options based on the mere consideration of their chronological age, without taking into account their biological fitness [134, 135].

**Fig. 5:**
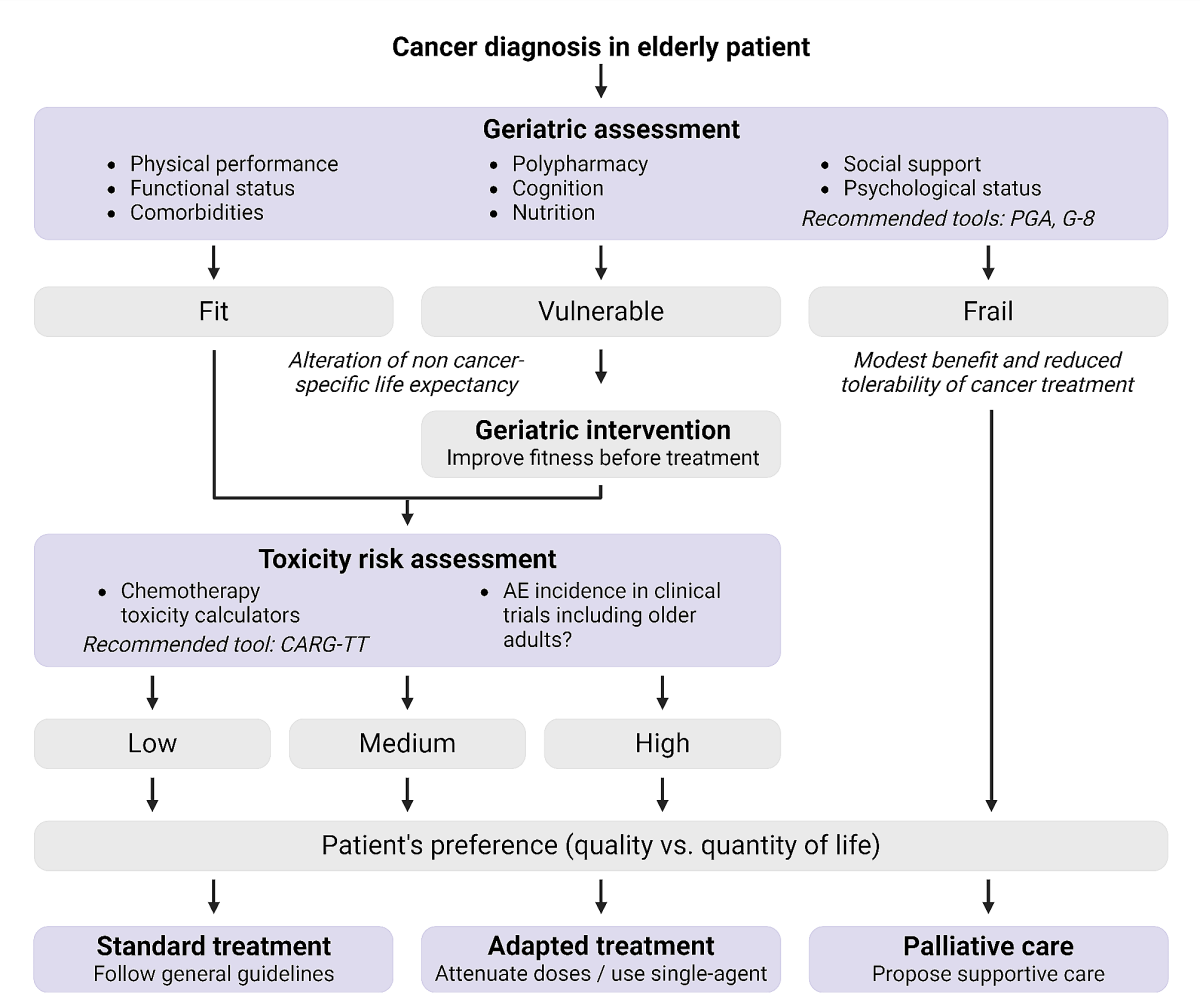
Practical management of geriatric patients after cancer diagnosis Flow chart for the adaptation of the general cancer clinical management guidelines to the specific needs of the geriatric population

To adapt cancer therapies to each cancer patient in a personalized fashion, recommendations have been formulated by American Society for Clinical Oncology [136], Federal Drug Administration [137], the Cancer and Aging Research Group [138], and the International Society of Geriatric Oncology Priorities Initiative [139]. This involves comprehensive geriatric assessment (GA) of patients before therapeutic decisions are made, ideally in the context of a medical team involving both geriatricians and oncologists. GA should include the combined evaluation of physical performance, functional status, comorbidities, polypharmacy, cognition, nutrition, social support, and psychological status [136]. GA then allows to classify patients into fit, vulnerable, and frail. Fit patients can be oriented towards standard of care, vulnerable individuals towards interventions that reduce geriatric conditions as they undergo adapted treatments (e.g., with reduced doses and number of cycles or giving preference to radiotherapy over chemotherapy), and frail persons towards palliative care [140] (Fig. 5). Randomized clinical studies demonstrated that GA can reduce serious toxic effects from cancer treatment [141]. Beyond GA, it is possible to measure biological parameters indicating health deterioration among older cancer patients such as the levels of circulating C-reactive protein, a parameter of systemic inflammation, to predict other parameters such as cognitive decline [142]. Indeed, it has been proposed to measure multiple parameters indicative of inflammation, cell senescence, telomere shortening, and epigenetic changes that may inform on the biological resilience of older cancer patients and then influence treatment decisions [143].

In synthesis, clinical oncology is confronted with the challenge of adapting treatments to a heterogeneous population of mostly elderly patients that differ in their biological and medical conditions. In this context, a major challenge is to transcend the idea that the extension of overall survival (quantity of life) constitutes the sole desirable endpoint and hence to consider the importance of quality-of-life as well [135].

## Conclusions

In this review, we have outlined some of the overarching principles governing the relationship between aging and cancer. Aging is strongly linked to cancer at three levels, namely, (i) because aging and oncogenesis share common mechanisms, (ii) because aging tissues favor tumor progression, and (iii) because tumor therapies undermine health and cause premature aging. Exceptions to these rules are constituted by (i) pediatric cancers that preferentially manifest during infancy rather than adulthood, (ii) the existence of progeroid syndromes without malignancies, and (iii) the fact that the oldest elderly exhibit a reduced incidence of new diagnoses of, and death from, cancer.

Worldwide estimations indicate that 1.6 billion individuals will be over 65 in 2050, implying a major surge in the number of age-related diseases including cancer. In this context, it will be important to decipher the precise mechanisms that link old age to the manifestation and progression of neoplasia and to develop broadly implementable strategies for the prevention, early detection and interception of malignant disease, hence avoiding the diagnosis of cancer at an advanced stage, when treatments become poorly tolerable, expensive, and mostly futile. Hence, investments in public and private research dealing with aging and cancer should be a priority for the future. Such investments will not only provide a molecular comprehension of the crosstalk between aging and malignancy, but will also lead to the identification of actionable targets for prophylactic or early-interceptive interventions on both processes.

It is reasonable to postulate that lifestyle interventions coupled to public policies designed to reduce exposure to industrial, nutritional, and environmental pollutants and to improve the economic and psychosocial status of the aging population, will allow to extend healthspan and to delay or avoid the manifestation of neoplastic disease. In this context, different countries have organized their pension and health systems, anti-pollutant strategies, as well as their focus on preventive versus curative medical interventions, in rather distinct ways. It will be a challenge for future investigation to perform carefully controlled inter-country comparisons so that the outcome of such policies can be accurately interpreted and improved. By applying policies that are successful in one country to others and by performing sophisticated performance measurements, it should be possible to perform large-scale multidisciplinary studies that will optimize a sustainable society that efficiently supports the prevention and interception of old age-associated cancer.

## Data Availability

No datasets were generated or analysed during the current study.

## Abbreviations

CAR: chimeric antigen receptor
CCS: childhood cancer survivor
CHIP: clonal hematopoiesis of indetermined potential
ECM: extracellular matri
GA: geriatric assessment
HGPS: Hutchinson-Gilford progeria syndrome
HAPLN2: hyaluronan and proteoglycan link protein 1
NK: natural killer
PDGF: platelet-derived growth factor
XP: xeroderma pigmentosum
